# Multiple Poses and Thermodynamics of Ligands Targeting Protein Surfaces: The Case of Furosemide Binding to mitoNEET in Aqueous Solution

**DOI:** 10.3389/fcell.2022.886568

**Published:** 2022-04-26

**Authors:** Linh Gia Hoang, Jonas Goßen, Riccardo Capelli, Toan T. Nguyen, Zhaoxi Sun, Ke Zuo, Jörg B. Schulz, Giulia Rossetti, Paolo Carloni

**Affiliations:** ^1^ INM-11, Forschungszentrum, Jülich, Germany; ^2^ Key Laboratory for Multiscale Simulations of Complex Systems, VNU University of Science, Vietnam National University, Hanoi, Vietnam; ^3^ IAS-5/INM-9, Forschungszentrum, Jülich, Germany; ^4^ Faculty of Mathematics, Computer Science and Natural Sciences, RWTH Aachen University, Aachen, Germany; ^5^ Department of Applied Science and Technology (DISAT), Politecnico di Torino, Torino, Italy; ^6^ College of Chemistry and Molecular Engineering, Institute of Theoretical and Computational Chemistry, Peking University, Beijing, China; ^7^ The Alexander Silberman Institute of Life Science, The Hebrew University of Jerusalem, Edmond J. Safra Campus at Givat Ram, Jerusalem, Israel; ^8^ Department of Physics, RWTH Aachen University, Aachen, Germany; ^9^ Department of Neurology, University Hospital Aachen (UKA), RWTH Aachen University, Aachen, Germany; ^10^ Jülich Supercomputing Centre (JSC), Forschungszentrum, Jülich, Germany

**Keywords:** NEET proteins, rational drug design, localized volume-based metadynamics, furosemide binding pose and affinity, furosemide, molecular dynamics, [2Fe-2S] cluster

## Abstract

Human NEET proteins, such as NAF-1 and mitoNEET, are homodimeric, redox iron-sulfur proteins characterized by triple cysteine and one histidine-coordinated [2Fe-2S] cluster. They exist in an oxidized and reduced state. Abnormal release of the cluster is implicated in a variety of diseases, including cancer and neurodegeneration. The computer-aided and structure-based design of ligands affecting cluster release is of paramount importance from a pharmaceutical perspective. Unfortunately, experimental structural information so far is limited to only one ligand/protein complex. This is the X-ray structure of furosemide bound to oxidized mitoNEET. Here we employ an enhanced sampling approach, Localized Volume-based Metadynamics, developed by some of us, to identify binding poses of furosemide to human mitoNEET protein in solution. The binding modes show a high variability within the same shallow binding pocket on the protein surface identified in the X-ray structure. Among the different binding conformations, one of them is in agreement with the crystal structure’s one. This conformation might have been overstabilized in the latter because of the presence of crystal packing interactions, absent in solution. The calculated binding affinity is compatible with experimental data. Our protocol can be used in a straightforward manner in drug design campaigns targeting this pharmaceutically important family of proteins.

## Introduction

**FIGURE 1:**
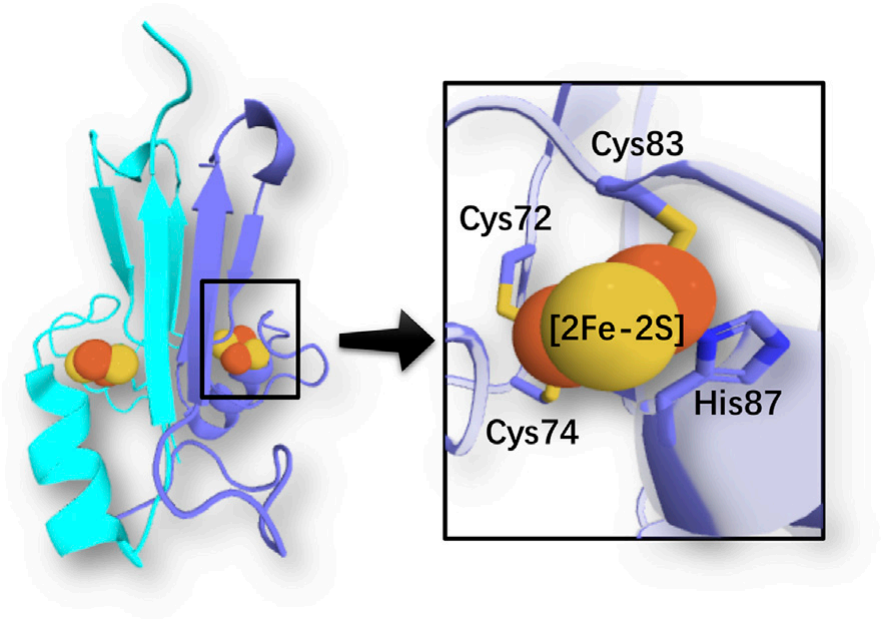
Coordination of an iron-sulfur cluster in a member of the NEET protein family (PDB ID: 2QH7 (Paddock et al., 2007)). Cartoon representation of chain A (light blue) and B (cyan). Sulfur and iron atoms are represented by yellow and orange spheres, respectively.

The human NEET [2Fe-2S] homodimeric proteins (such as mitoNEET (Colca et al., 2004; Paddock et al., 2007) and NAF-1 (Conlan et al., 2009)) have emerged as important targets for pharmaceutical intervention, from cancer and diabetes, to metabolic and neurodegenerative diseases (Nechushtai et al., 2020). These proteins are located on the outer membrane of mitochondria and mitochondria associated membranes, and, in the case of NAF-1, also on the endoplasmic reticulum’s membrane. Each subunit features a 3Cys:1His coordinated [2Fe-2S] cluster (Figure 1), either in a reduced (Fe(III)-Fe(II)) or oxidized (Fe(III)-Fe(III)) state. In the reduced state, the ferrous ion is located close to the protein surface and bound to the histidine (Dicus et al., 2010) (Figure 1). The clusters are reduced and inert in physiological conditions. Oxidation under oxidative stress leads to a cluster-labile oxidized state: the cluster can then be released or transferred to apo-acceptors (Landry and Ding, 2014). Cancer cells may express more human NEET proteins than healthy ones to support their required high level of mitochondrial iron and reactive oxygen species (Darash-Yahana et al., 2016). In contrast, cells undergoing neurodegenerative or metabolic disease express less or no human NEET proteins (Kusminski et al., 2016; Nechushtai et al., 2020). Thus, drugs regulating the [2Fe-2S] cluster stability of human NEET proteins might be able to counteract cell derangement associated with many diseases.

So far, a few ligands targeting mitoNEET (Colca et al., 2004; Paddock et al., 2007) and human NAF-1 (Conlan et al., 2009) have been identified. They have been shown to affect cluster release *in vitro,* and to bind in their cluster binding domain (Geldenhuys et al., 2019; Marjault et al., 2021). Efficient computational protocols predicting poses and affinities of ligands would be of paramount importance to improve the potency of such drug leads. They allow for artificial intelligence-based screening of new compounds, with optimal solubility and selectivity (Adeshina et al., 2020; Gao et al., 2020). In addition, they provide an estimation of ligands affinities for the oxidized human NEET proteins, which is very useful as accurate *in vitro* measurements of such affinities may at times be challenging because of the high liability of the cluster at acidic pH (Zuo et al., 2021).

Docking approaches, currently used in the design of ligands targeting enzymes and receptors binding sites, may encounter difficulties here. Indeed, they do not accurately estimate all the possible interaction and desolvation contributions of ligands targeting proteins which lack well-defined binding pockets (Deng et al., 2015). Thus, docking of small molecules on the flat/shallow binding sites of these proteins may lead to false-positives (Li et al., 2014; Guterres and Im, 2020). This problem can be even more exacerbated in transition metal-based systems (Chen et al., 2007), like the NEET proteins.^1^

Both problems were addressed in the past by some of us by 1) developing molecular simulation docking protocols on proteins lacking specific pocket definitions (Kranjc et al., 2009); and 2) by parameterizing both oxidized and reduced NEET [2Fe-2S] clusters for molecular simulations (Pesce et al., 2017; Zuo et al., 2021). Here, by capitalizing on this work, we use a variant of well-tempered metadynamics (WT-MetaD) enhanced sampling simulations (Barducci et al., 2008) to predict the pose and the potency of the ligand targeting mitoNEET. WT-MetaD is an exact method to calculate the free energy of binding as a function of collective variables (CVs) (Barducci et al., 2008). This variant is the so-called Localized Volume-based (LV) MetaD. This approach has already been successfully applied to study ligand binding to proteins with very high computational efficiency (Zhao et al., 2021).

**CHART 1:**
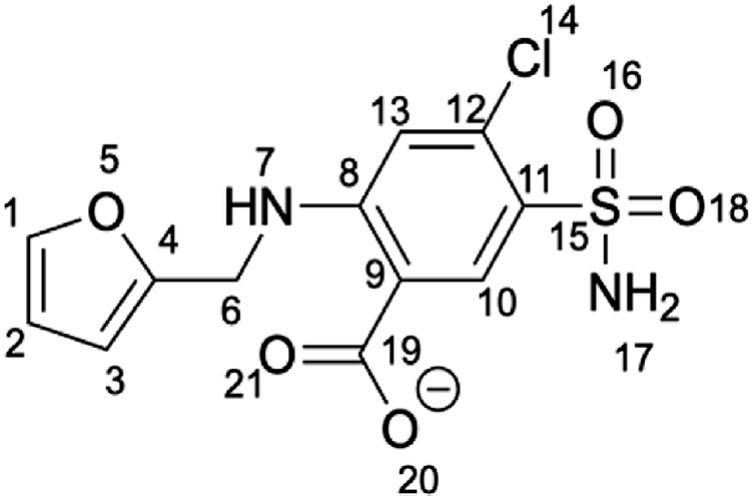
Structure of furosemide in its most probable protonation state at pH 7.

**FIGURE 2:**
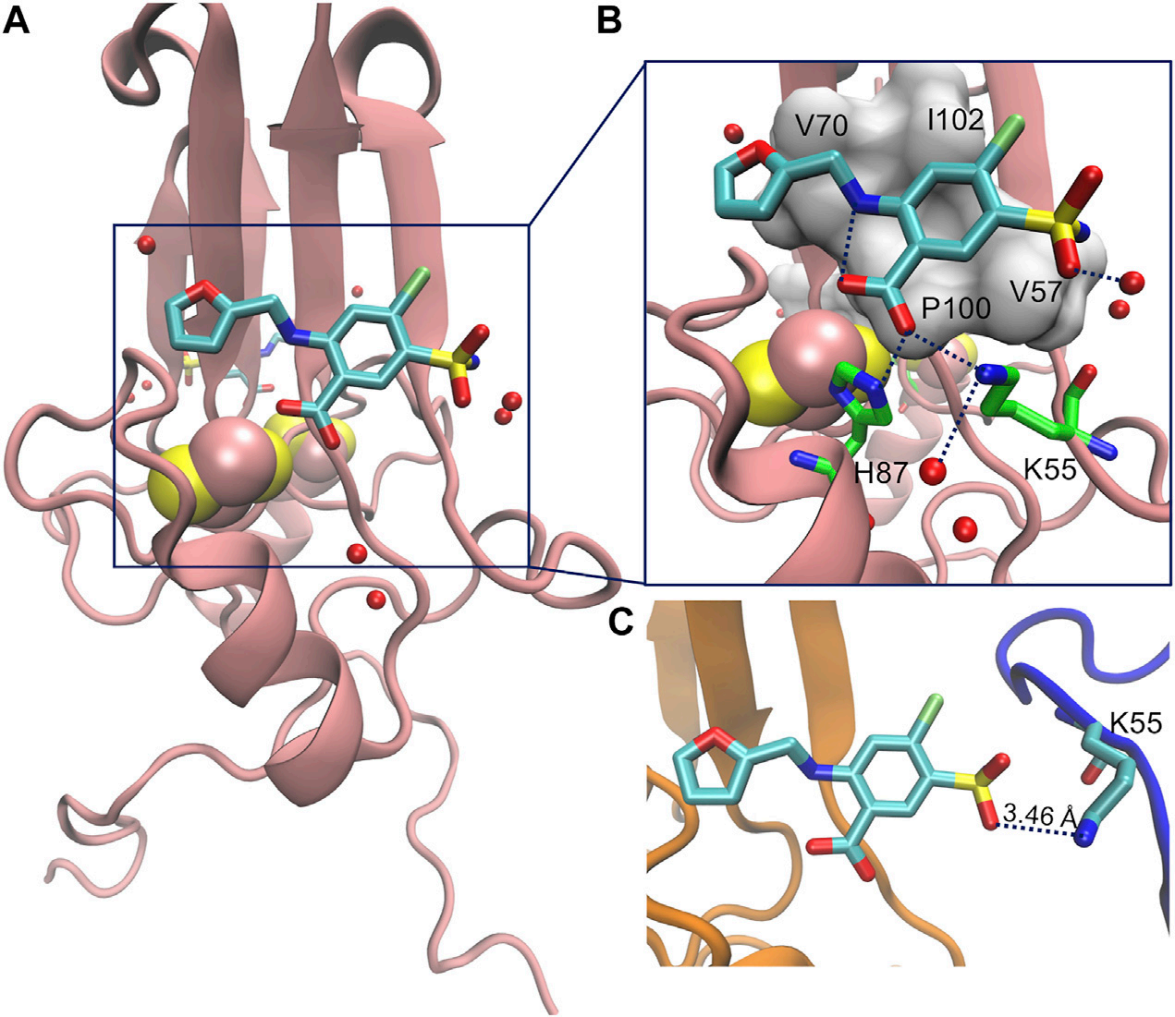
**(A)** Crystal structure of ligand furosemide binding to mitoNEET protein at pH 7.0. **(B)** Close up showing furosemide-protein H-bonds/salt bridges interactions. **(C)** Interactions of the ligand with the protein image (in blue color) in the crystal.

We focus on the furosemide (4-Chloro-2-[(furan-2-ylmethyl)amino]-5-sulfamoylbenzoic acid) molecule (Chart 1), which slows down cluster release *in vitro,* and its binding to mitoNEET in the oxidized state (Geldenhuys et al., 2019). This is the only ligand/human NEET protein complex deposited on the protein data bank so far (Geldenhuys et al., 2019). Affinity measurements by radioligand displacement (Geldenhuys et al., 2016) are also available. The X-ray structure shows that the ligand binds in a shallow binding pocket located at the interface between the cluster and the upper part of the monomer (Figure 2A). Specifically, the ligand’s carboxyl group forms hydrogen bonds (H-bonds) with the iron bound histidine residue (H87) of one subunit and a lysine (K55) from the other (Figure 2B). The benzene ring forms hydrophobic interactions with V57, P100, I102, while the furan ring with V70. The NH group forms an intramolecular H-bond with the carboxyl group of the ligand. Finally, the sulfonamide group also forms an H-bond with the protein from the adjacent asymmetric unit (Figure 2C).

Our simulations provide a quantitative estimation of the affinity of binding, which is not too dissimilar from experiment. Most importantly, we suggest, based on our calculations, that furosemide can actually bind in several binding poses around the same surface pocket, including the one observed in the crystal structure. The latter may be stabilized by crystal packing interactions in the solid state (Figure 2C), as observed before (Marelli et al., 2014). These interactions are absent in water solution (Kranjc et al., 2009; Arif et al., 2011; Geldenhuys et al., 2019).

## Materials and Methods

### System Preparation

The crystal structure of furosemide binding to mitoNEET protein was downloaded from the Protein Data Bank (PDB ID: 6DE9) (Geldenhuys et al., 2019). Maestro (Sastry et al., 2013) (VERSION 2017-2) and GROMACS/2019.4 (Lindahl et al., 2001; Abraham et al., 2019) patched with Plumed 2.5 (Jakalian et al., 2002; Bonomi et al., 2019) were used to perform preparation steps. For protein, water, ions and [2Fe-2S] clusters, we used the AMBER ff99SB-ILDN-DEP (Lindorff-Larsen et al., 2010), TIP3P (Jorgensen et al., 1983), the Åqvist potential (Ȧqvist, 1990) and force field parameters calculated in our previous work (Pesce et al., 2017), respectively. The ligand was parameterized using the General AMBER Force Field (Wang et al., 2004) obtaining the single-point charges using the semi-empirical AM1-BCC method (Jakalian et al., 2002) generated by the acpype utility script (Sousa da Silva and Vranken, 2012) (Supplementary Figure SI1). The system with protein and ligand was solvated in a periodic octahedron box with 28,008 TIP3P (Jorgensen et al., 1983) water molecules. Finally, counterions Na^+^ (80) and Cl^−^ (87) were added to neutralize the system and mimic the physiological salt concentration at 150 mM. The distance from the protein to the edge of the box turned out to be 20 Å or more during the simulations, avoiding self-interaction artifacts.

The bonds were constrained using the LINCS algorithm (Hess et al., 1997). The smooth Particle Mesh Ewald method (Essmann et al., 1995) was used to treat the long-range electrostatic interactions, with a grid spacing value of 1.2 Å. The cutoff for short-range electrostatic interactions and van der Waals was set to 14 Å. The temperature and pressure of system (T = 298 K, *p* = 1 bar) were controlled using the Nose-Hoover thermostat (coupling the system every 0.2 ps with a chain length of 10) (Evans and Holian, 1985) and isotropic Parrinello−Rahman barostat (coupling the system every 0.5 ps with a compressibility of 4.5.10^–5^ bar^−1^) (Parrinello and Rahman, 1981), respectively. The integration step was set to 2 fs.

### Molecular Simulations

We performed energy minimization to the system with the steepest descent algorithm, setting the converge criteria to 2.4 kcal mol^−1^ nm^−1^ of the maximum force (Haug et al., 1976). Then, we gradually heated the system in 40 points to 298 K in 1 ns of annealing (Supplementary Figure SI2). The system underwent the first 5 ns NVT Molecular Dynamics (MD) at 298 K with a harmonic restraint of 240 kcal mol^−1^ nm^−2^ on both furosemide and protein to maintain the initial experimental conformation. All the bonds were constrained with the LINCS algorithm (Hess et al., 1997). Then, 75 ns NPT MD were performed. Next, the free energy landscape associated with furosemide binding to the protein was investigated by Localized Volume-based Metadynamics (LV-MetaD).

LV-MetaD is a WT-MetaD (Barducci et al., 2008) protocol where a history-dependent potential (called bias) is deposited on three apt collective variables (CVs), *i.e.,* a convenient representation of the reciprocal position of the furosemide with respect to the host protein. To minimize convergence time, the furosemide is constrained in a limited (localized) volume close to the binding pose observed in crystal structure *via* the imposition of a restraining potential. The coordinate system used to represent the furosemide position in the reference frame of the host protein depends on the shape of the restraining potential. Here, we used a parabolic solid volume restraining as in the original implementation of the method (Zhao et al., 2021). The collective variables were: ρ, defined as the distance between the center of mass of the furosemide and the protein, τ, the parameter that defines the parabolic-solid shape of the volume (Zhao et al., 2021), θ, defined as the azimuthal angle of its orthogonal projection on the x-y plane (Supplementary Figure SI3). To guarantee a correct sampling for both the bound and the unbound state, we limited the restraining volume (and thus the CVs ranges) to include 1) the binding pose observed in crystal structure, 2) the neighboring regions, and 3) enough volume to observe the ligand being completely solvated (Supplementary Figure SI4). The protein-furosemide axis was aligned to the *x*-axis in our system. To avoid artifacts associated with periodic boundary conditions, we applied a restraining bias that kept the protein’s center of mass to 10 Å or less from the simulation box center. To avoid unfolding problems due to volume bias, the protein backbone atoms, which are not inside the volume, were restrained to their initial positions so that the overall RMSD was smaller than 3 Å.

We applied the bias potential on the system along the defined CVs, setting the initial height of Gaussian hills to 0.287 kcal/mol and deposited every 1 ps. The Gaussian widths are 1 Å, 0.04, and pi/8 for ρ, τ, θ, respectively. The bias factor was chosen to be 20. LV-MetaD for 650 ns. The last 100 ns trajectory was used for the reweighting procedure using the Tiwary-Parrinello estimator (Tiwary and Parrinello, 2015). The reweighting procedure allows us to compute the projection of the free energy landscape as a function of apt order parameters that define clearly the bound and unbound states. From this last free energy surface, it is possible to obtain furosemide’s binding free energy. In our case, we choose to consider two variables: the distance between the protein and the furosemide centers of mass, and the number of H-bonds between the furosemide and the residues inside the volume, defined using the following switching function:

$$s_{ij}=\frac{1-{\left(\frac{r_{ij}-d_{0}}{r_{0}}\right)}^{n}}{1-{\left(\frac{r_{ij}-d_{0}}{r_{0}}\right)}^{m}}$$

Here we have *n* = 8 and *m* = 12, d_0_ was set to 0 and r_0_ = 2.5 Å. The number of H-bonds is ∑s_ij_. To evaluate the errors of the free energy, we used a block average analysis (Supplementary Figure SI5). In the lowest energy basin, each pose was equilibrated by 30ns of unbiased MD. MD and LV-MetaD simulations were carried out by GROMACS/2019.4 (Lindahl et al., 2001; Abraham et al., 2019) patched with PLUMED-2.5.2 (Tribello et al., 2014; Bonomi et al., 2019).

## Results and Discussion

The identification of ligand poses on NEET proteins may require approaches that go beyond straightforward molecular docking, as the ligand binds on the protein surface (and not to a binding site), close to a multinuclear iron site. Here we have used enhanced sampling methods to predict poses and affinity of furosemide (Chart 1) to the mitoNEET protein, similarly to what done by some of us in the case of a ligand binding to the surface of the prion protein, where the accuracy of our prediction was established by a comparison with NMR data (Kranjc et al., 2009). Our computational protocol profits also from an apt parametrization of the metal cluster recently developed by some of us (Pesce et al., 2017; Zuo et al., 2021).

Our protocol has involved 75 ns of molecular dynamics (MD, Supplementary Figure SI6) starting from the X-ray structure of oxidized mitoNEET in complex with furosemide (Figure 2A). After a short simulated annealing procedure, the system was brought to the same conditions as the *in vitro* assays. The MD calculations are followed up by Localized Volume-based Metadynamics (Zhao et al., 2021) enhanced sampling method. These predict the free energy of furosemide unbinding in the canonical ensemble as a function of three apt collective variables (Supplementary Figure SI3 and Methods for details). The simulations converged after 600 ns (see Supplementary Figures SI7, SI8). With the reweighting procedure (Tiwary and Parrinello, 2015), we find it convenient to plot the free energy as a function of the distance *d* of the centers of mass of the furosemide and of the [2Fe-2S] cluster, as well as the number *N* of furosemide/protein H-bonds and salt bridges.

**FIGURE 3:**
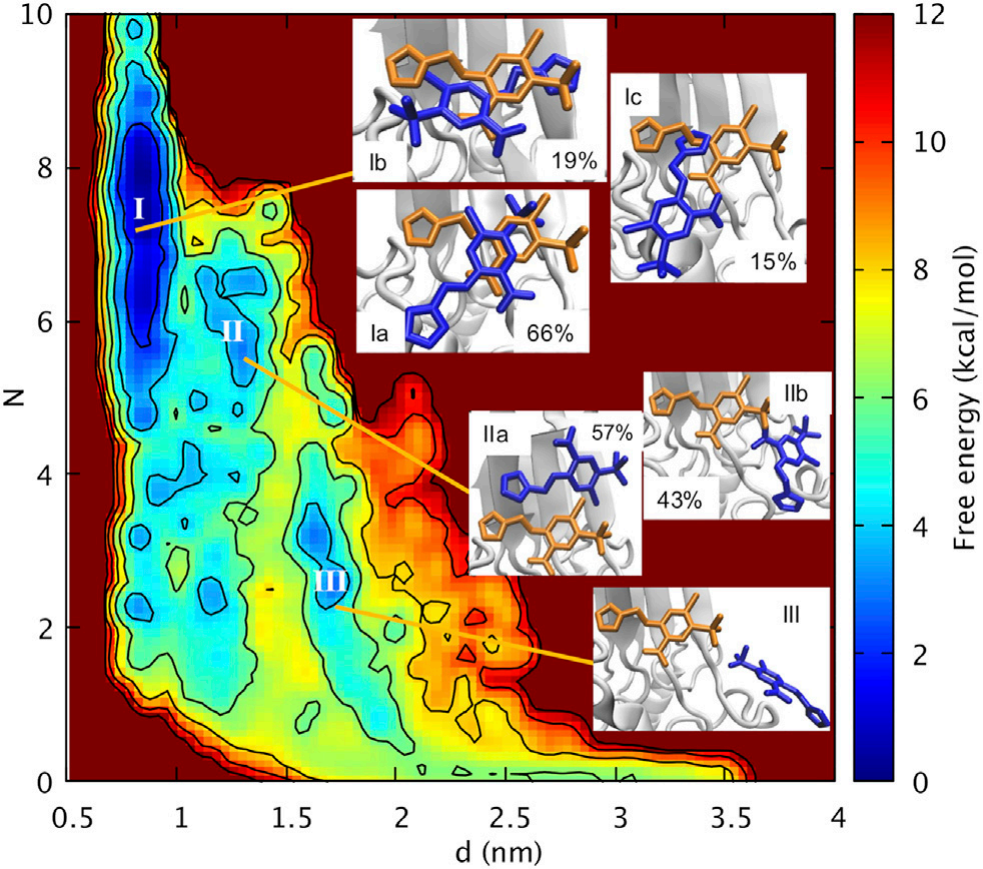
Free energy of furosemide unbinding as a function of the distance between the centers of mass of the furosemide and of the Fe-S cluster (*d*) and the number of H-bonds/salt bridges (*N*)*.*

**FIGURE 4:**
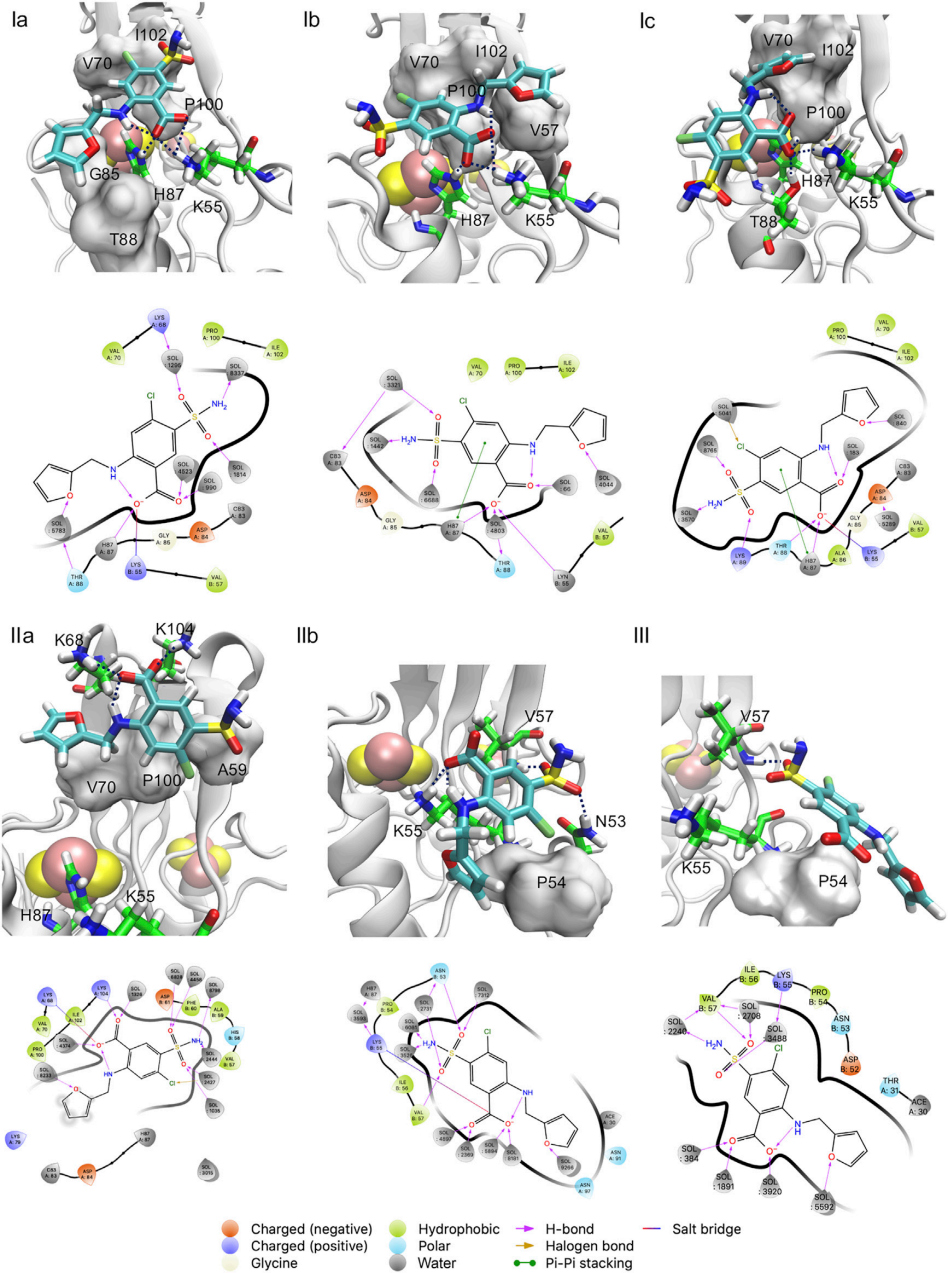
**(Ia–c, IIa,b, III**) poses of Figure 2. Both the 3D structure and the ligand-protein interaction diagram are shown. H-bonds/salt bridges are drawn as dashed lines in the 3D structures.

Basin **I** is the absolute minimum, lower than about 2 kcal/mol than the local minima **II** and **III.** In **I**, the ligand features three poses with diverse orientations (**Ia-c).** In each pose the ligand is rather close to the cluster (0.77 nm < *d* < 0.95 nm) and exhibits extensive intramolecular interactions (5 < *N <* 9, Figure 3). This includes the salt bridge between the ligand and Nζ@K55 and the H-bond with Nε@H87 (Supplementary Table SI2) (Figure 4), present also in the X-ray structure (Geldenhuys et al., 2019). However**,** in **Ia-b,** the salt bridge involves both oxygen atoms and not only one atom as in the X-ray structure (Figure 2), and in **Ic,** the furosemide’s carboxyl group forms a H-bond with T88 side chain. In all the minima shown here, the carboxy-NH intramolecular H-bond is maintained.

The orientations of the aromatic rings and the interactions of the sulfonamide group with the protein differ from those of the X-ray structure.

In **Ia**, by far the most populated conformer^2^, the sulfonamide group forms a water mediated H-bond with Nζ@K68 (Supplementary Table SI2), while, as discussed above, it interacts with the protein from the adjacent asymmetric unit in the X-ray structure. The furan ring replaces its hydrophobic interactions with V70, present in the X-ray structure, with those with G85 and T88 (Supplementary Table SI2); the benzene ring, while keeping its hydrophobic interactions with P100, I102, replaces the interactions with V57 with those with V70 (Supplementary Table SI2). In **Ib**, the furan ring interacts with V57 and I102, while the benzene ring interacts with V70 and P100 and it also forms a π-π stacking interaction with H87 (Supplementary Table SI2 **)**. The sulfonamide and the carboxyl groups form water-mediated H-bonds with the C83 backbone unit^3^ and the T88 side chain, respectively (Supplementary Table SI2). In **Ic**, the furan moiety forms hydrophobic contacts I102, V70, P100, the benzene ring is solvent-exposed.

30 ns MD starting from **Ia-c** shows that 1) binding poses **Ib-c** are transient and can interconvert into each other within a few ns (Supplementary Figure SI9). 2) **Ia** samples other orientations including the one found in the crystallographic pose (Supplementary Figure SI10), and this binding pose reproduces also the experimental electronic density (Supplementary Figure SI11E).^4^ This variability results from the very shallow binding site as found in the mitoNEET and is already hinted at by challenges in resolving the electron density around the ligand’s furan moiety (Supplementary Figure SI11A)^5^. The discrepancy between the presence of a unique binding pose and an ensemble of poses (including the X-ray one) in the simulations is attributed here to a packing effect in the crystal. Indeed, in the periodic system (crystal structure), the ligand features a H-bond with K55 of an image protein and this interaction obviously does not present in water solution. We can expect therefore that this interaction stabilizes a specific conformation, following the conformational selection hypothesis (Nussinov et al., 2014), while in water solution an ensemble of conformations may be present.

The free energy of binding/unbinding (7.7 ± 0.8 kcal/mol), from basin **I** to the fully solvated ligand is not too dissimilar from the experimental free energy of binding at the same temperature (5.8 kcal/mol) (Supplementary Table SI3 **)**.

Basin **II** is located a bit farther from the cluster than **I** (1.1 nm < d < 1.4 nm, Figure 3). It forms a smaller number of polar intermolecular interactions (5.2 < *N <* 6.7). It features two similarly populated poses (**IIa,b,** Figure 4). The H-bond between the carboxyl group and H87 is replaced by a salt bridge with K68 (in **IIa)** or by an H-bond with the solvent (in **IIb).** The salt bridge with K55 is maintained only in **IIb.** In **IIa,** it involves K104. The sulfonamide group forms H-bonds with V57 backbone and N53 in **IIb**. The furan ring forms hydrophobic interactions with V70 (**IIa**) and P54 (**IIb**), while the benzene ring with A59, I102 (**IIa**), V57 side chain (**IIb**). The aromatic rings are more solvent exposed than those in **I.** The higher solvation of the furosemide may account, at least in part, for the higher free energy of this minimum.

Basin **III** is located farther from the cluster than **II** (1.5 nm < *d* < 1.8 nm). It has lost all the intermolecular interactions in **I-II** (2.3 < *N <* 3.5, Figure 3). The carboxyl group is fully hydrated, while the sulfonamide forms direct and water-mediated H-bonds with V57 as well as a water-mediated H-bond with K55 (Figure 4). The aromatic rings have hydrophobic contacts with only P54 and are more solvent-exposed than basin **I** and **II**.

In conclusion, our simulations reproduce the pose of the X-ray structure (Supplementary Figure SI9B) and the experimental electronic density (Supplementary Figure SI11E), suggesting that this is only one among an ensemble of structures in water solution. The binding free energy values are quantitatively close to the experimental data. Thus, our paper is consistent with the available experimental data.

## Conclusion

Here, we have investigated furosemide binding to mitoNEET in the oxidized state with the following goals in mind: 1) the comparison with the X-ray structure, which is in the oxidized state (Geldenhuys et al., 2019) and 2) to present an advanced computational approach able to investigate, for the first time to the best of our knowledge, quantitatively ligand binding to human NEET proteins, a highly important pharmacological target. Our study suggests that the ligand binds to several isoenergetic poses in water solution, including the one emerging from the X-ray structure. The latter pose is likely to have been selected because of crystal packing interactions. The calculations provide an estimate of the affinity which is fully compatible with that experimentally determined. Driven by the computational findings here, NMR and/or site-directed mutagenesis experiments in the binding regions, such as in those in (Kranjc et al., 2009) and (Zhou et al., 2010) for other ligands bound to protein surfaces, would be additional validations of our calculations.

Our protocol is very general and it emerges as a useful tool to predict binding affinity and multiple poses of ligands targeting human NEET proteins.

## Data Availability

The raw data supporting the conclusion of this article will be made available by the authors, without undue reservation.

## Footnotes

1 In spite of these limitations, successful applications of simplified docking approaches such as MAD-28 to mitoNEET/NAF-1 have been reported (Bai et al., 2015). These applications are reported in the SI for furosemide binding to mitoNEET, allowing for a comparison with the free energy calculations performed in this work (Supplementary Table SI1).

2 The populations for **Ia-c** are 66%, 19%, 15%, respectively.

3 This cysteine is bound to the iron atom close to the solvent.

4 For a direct comparison between X-ray and basins poses see Supplementary Figure SI12.

5 The populations for **IIa** and **IIb** are 57% and 43%, respectively
