# Supplementary material for: Multiple Poses and Thermodynamics of Ligands Targeting Protein Surfaces: The Case of Furosemide Binding to mitoNEET in Aqueous Solution
Source: Front Cell Dev Biol. 2022 Apr 26;10:886568. doi: 10.3389/fcell.2022.886568 (PMC9086288; doi:10.3389/fcell.2022.886568)
Supplement: Supplementary file 1 [file DataSheet1.pdf]

Supplementary Material

1 Supplementary Information

1.1 Localized Volume-Based Metadynamics Entropic Correction

To remove the entropic contribution to binding free energy in LV-MetaD, it is necessary to apply a correction to the final free energy difference (Capelli et al., 2019). In particular, we have:

$$\Delta G^0 = \Delta G_{MetaD} + \Delta G_{corr}$$

$$= \Delta G_{MetaD} \;+\; RT\; log(\frac{V^0}{V_{LV} - V_{prot}})$$

Where ΔG_0 is the standard binding free energy, ΔG_{MetaD} is the free energy difference computed via LV-MetaD, and ΔG_{corr} is the correction term. The latter is basically given by the concentration difference between the standard concentration (V^0 is the standard volume, 1660 Å^3) and the volume accessible to the ligand in the localized volume (V_{LV} is the localized volume, and V_{prot} the portion of such volume occupied by the protein).

1.2 Molecular Mechanics-Generalized Born Surface Area (MM-GBSA)

Prior to the docking simulations, we retrieved the protein structure of mitoNEET (PDB: 6DE9) as a homodimer from the Protein Data Bank (Geldenhuys et al., 2019). We used the standard parameters for refinement implemented within the Protein Preparation Wizard (Schrödinger, 2019b). Furosemide was prepared using the ligprep utility program implemented in Schrödinger 2019-4 (Schrödinger, 2019a). The pH of 7.0±2.0 was chosen and the other standard parameters setting as default. We chose the centroid of the furosemide bound to mitoNEET as the centroid of the 20x20x20 Å^3 bounding box. according to (Bai et al., 2015). The van der Waals radius and charge scaling factors were set to 1.0 during grid generation. The docking was performed in Schrödingers' glide SP mode with at most 200 top-scoring binding modes were reserved for post docking minimization. (Halgren et al., 2004; Sastry et al., 2013) All settings were set to default, i.e. with the switches of the ligand flexible and the ring conformation sampling turning on, and the scaling factor of van der Walls radii as well as the partial charge cutoff for the ligand atoms setting as 0.8 and 0.15 respectively. We calculated the energies for the refined binding modes with the MM-GBSA calculation module in Maestro 2019-4 using the OPSL-2005 force field and the flexible distance set to 20 Å (Shivakumar et al., 2010).

2 Supplementary Figures and Tables

2.1 Supplementary Figures

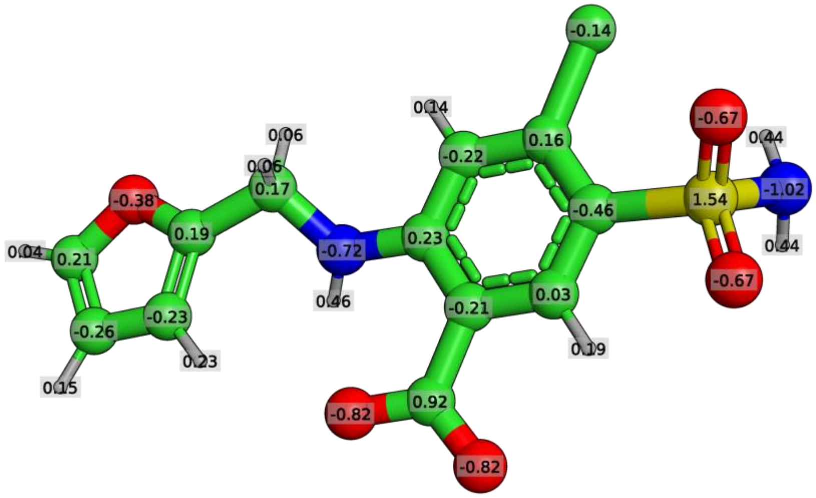

Figure SI1. The ligand was parametrized using the Generalized AMBER Force Field (GAFF) (Wang et al., 2004) obtaining the single-point charges using the semi-empirical AM1-BCC method.

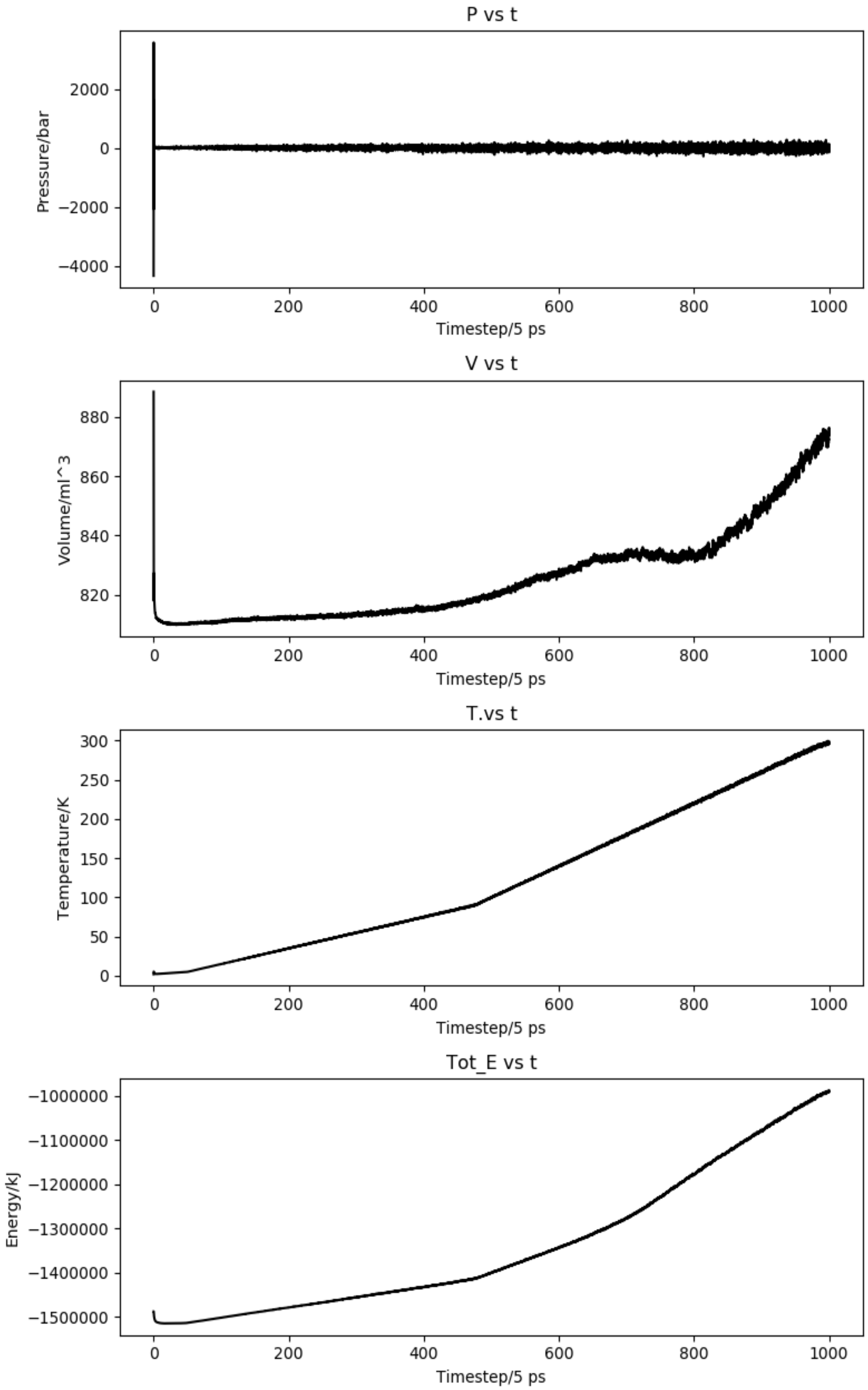

Figure SI2. Simulated annealing protocol. Line plots for pressure, volume, temperature and total energy while heating the system to 298 K in 1 ns are shown.

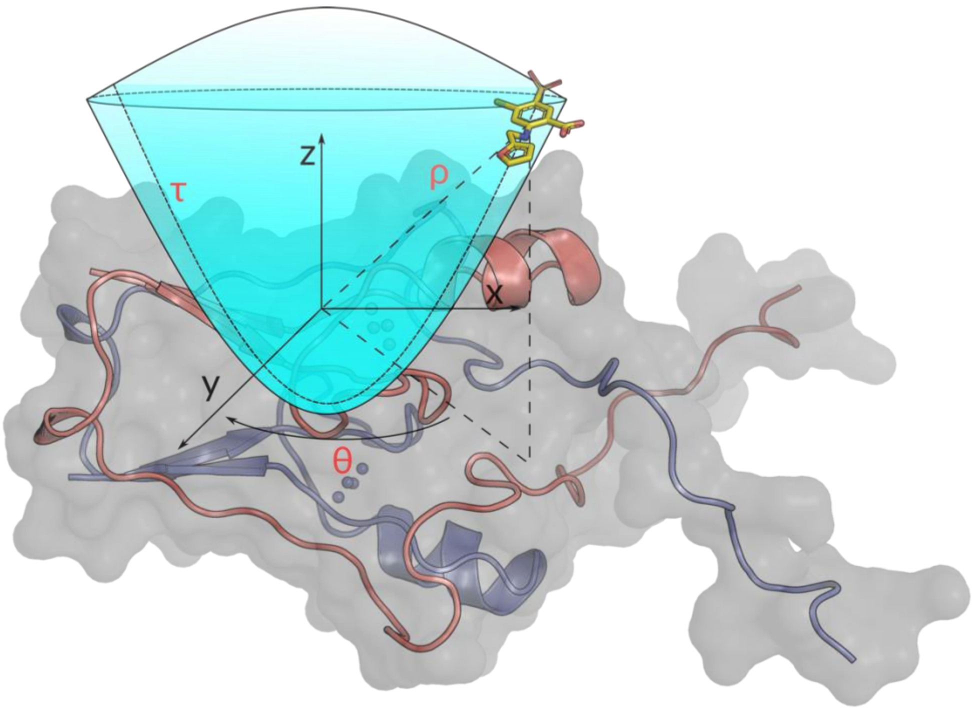

Figure SI3. Localized Volume-based Metadynamics CVs definition: ρ, defined as the distance between the center of mass of the ligand (ligand visualized with yellow sticks) and the protein (red and blue cartoon representation), τ, the parameter that defines the parabolic-solid shape of the volume (Zhao et al., 2021), θ, defined as the azimuthal angle of its orthogonal projection on the x-y plane.

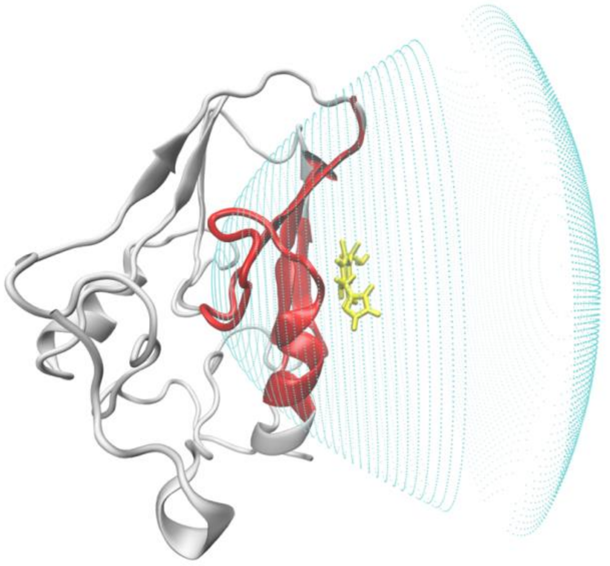

Figure SI4. The restraining volume which includes the binding pose observed in crystal structure (yellow) and the neighboring regions (red).

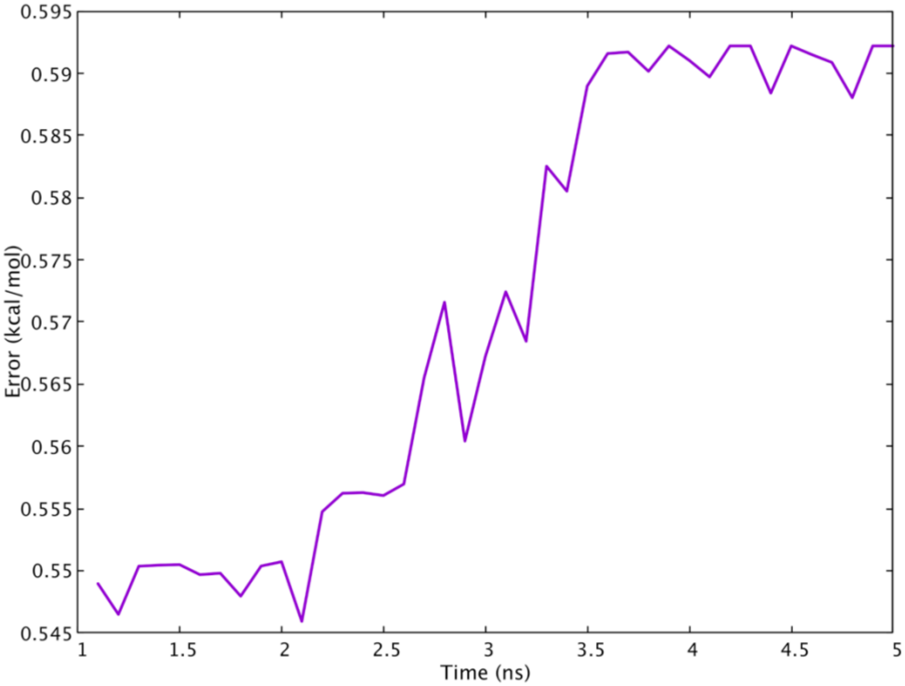

Figure SI5. Error calculation for the FES by means of block averaging. The error is represented as a function of the size of the blocks.

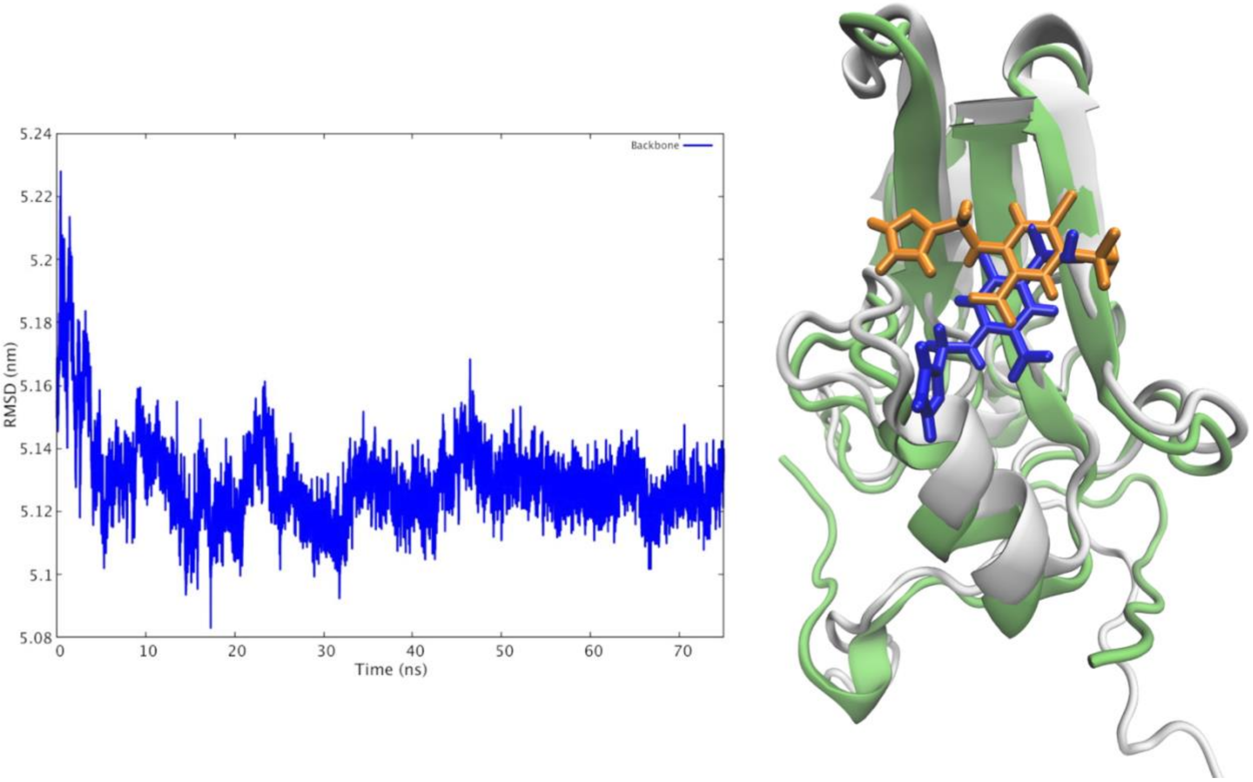

Figure SI6. Left: RMSD of protein backbone shows convergence after 75 ns of MD. Right: Comparison between the last MD snapshot and the X-ray structure (Last MD snapshot: protein in green, ligand in blue; X-ray structure: protein in white, ligand in orange).

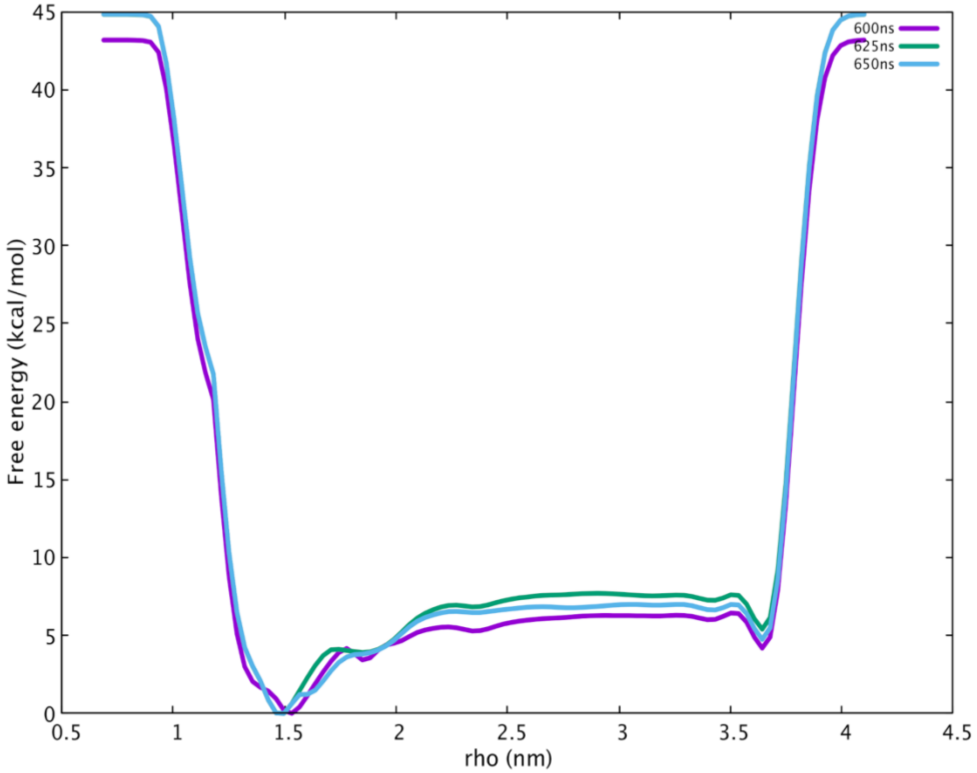

Figure SI7. Free energy surfaces are projected along the distance between the center of mass of the protein and the center of mass of the ligand at 600, 625 and 650 ns of simulation time. The position of the minima and the profile appear converged.

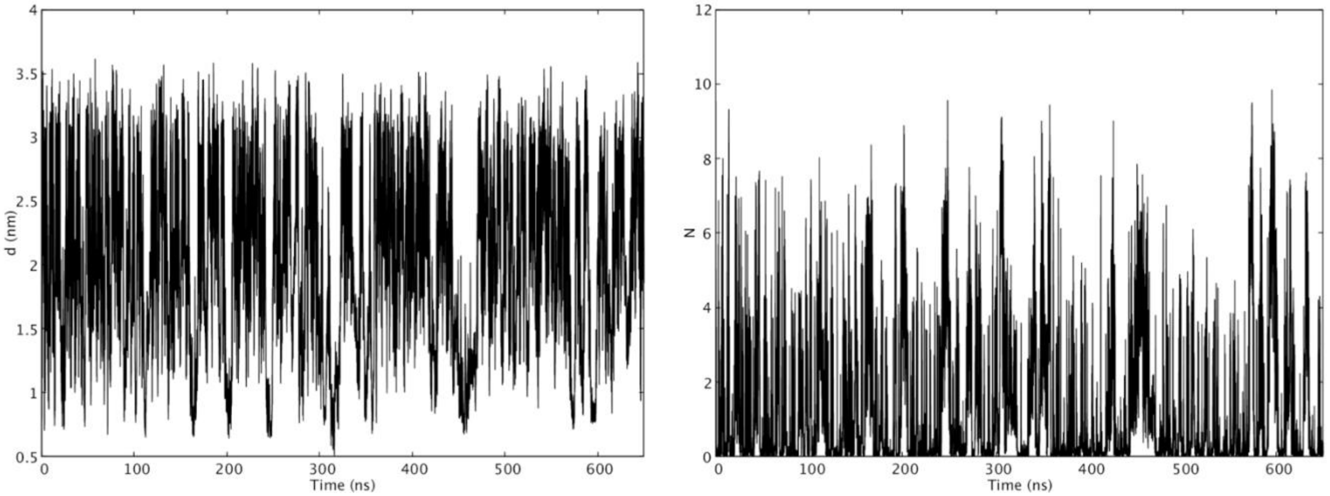

Figure SI8. Time series for the two CVs (distance from the cluster on the left and number of H-bonds/salt bridges between ligand and protein on the right) along which we projected the FES. We can observe a diffusive behavior in the CV space that corroborates the idea that the simulation converged.

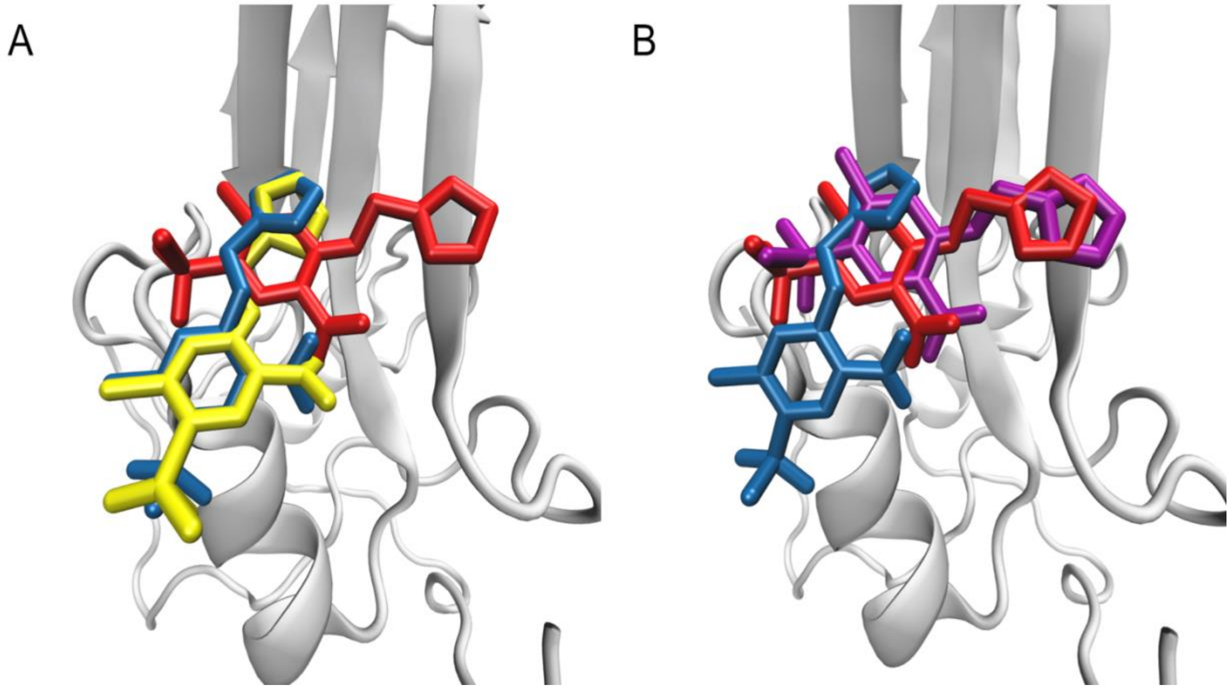

Figure SI9. The interconversion of pose Ib and Ic of basin I from unbiased MD simulations starting from each pose. **(A)** Pose **Ib** (in red), pose **Ic** (in blue) and a frame from MD of pose **Ib** that is similar to pose **Ic** (in yellow). **(B)** Pose **Ib** (in red), pose **Ic** (in blue) and a frame from MD of pose **Ic** that is similar to pose **Ib** (in purple).

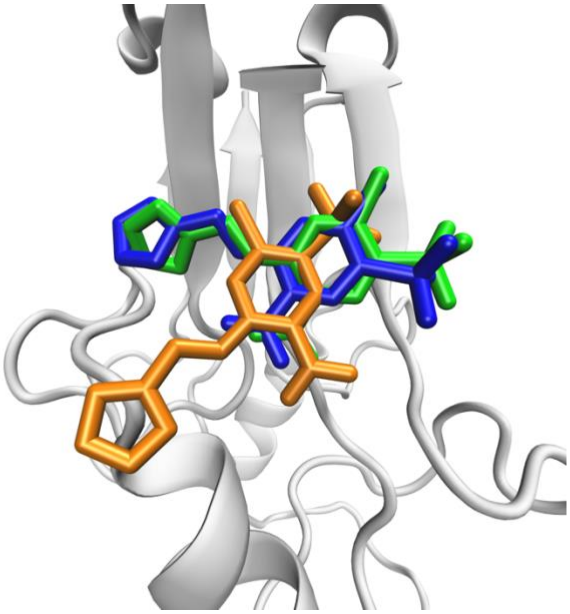

Figure SI10. Pose **Ia** (in orange), **crystallographic pose** (in blue) and a frame from MD of pose **Ia** that is similar to **crystallographic pose** (in green)

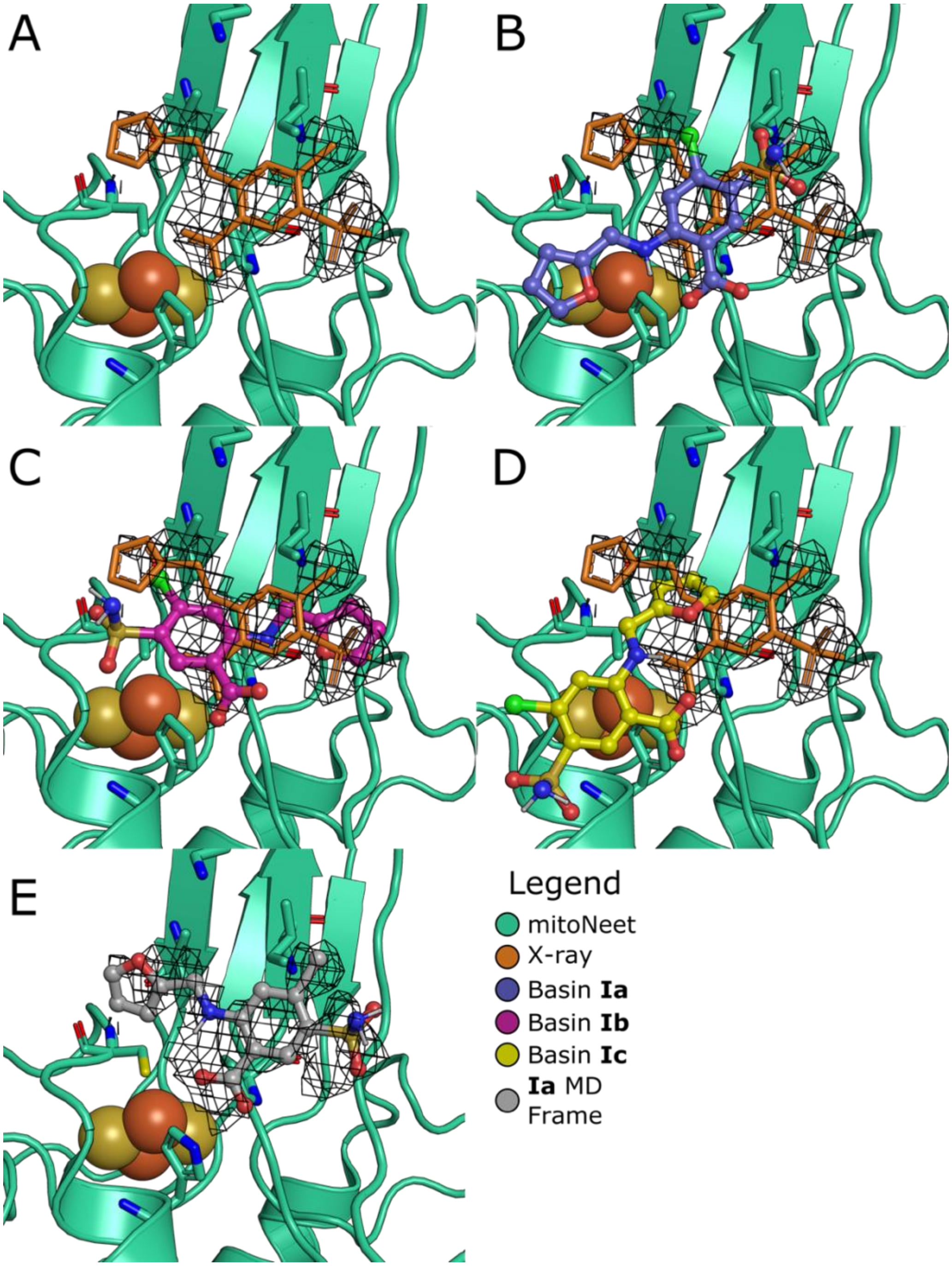

Figure SI11. Overlap of pose **Ia-c** (B-D) structures of furosemide (blue, magenta, yellow, shown as ball and sticks) with crystal structure of mitoNEET (A, green, comic, PDB ID 6DE9) and 2Fo–Fc map (contoured at 0.8 σ as black wireframe, reported in (10.2210/pdb6de9/pdb) showing the experimentally-derived electron density of furosemide. The electronic density of the furan moiety is not resolved (Geldenhuys et al., 2019). (E) Comparison of furosemide's pose in the MD frame that best reproduces the X-ray structure (**Ia**, grey, ball and stick, see Text) with the 2Fo–Fc map. The figure shows that this binding pose reproduces also the experimental electronic density similarly to the pose of the X-ray structure.

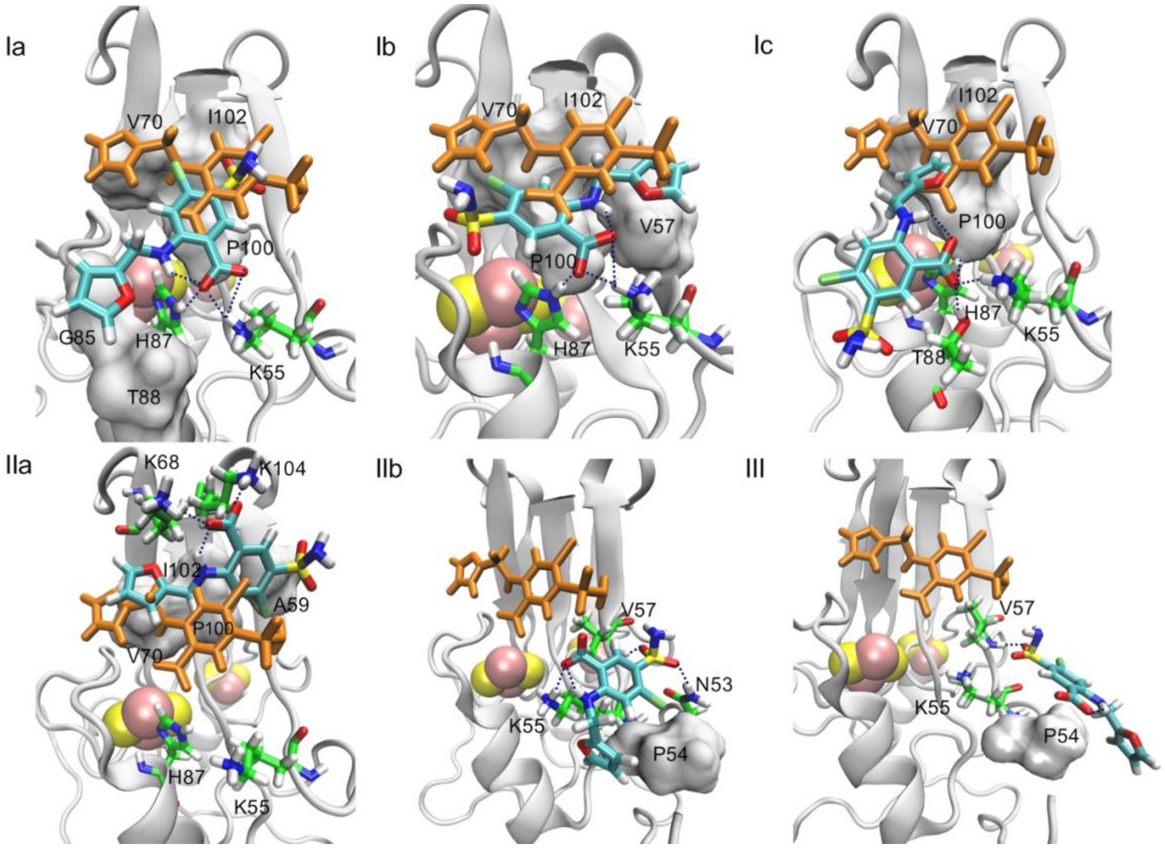

Figure SI12. Ia-c, IIa-b, III poses presented in Figure 2**.** H-bonds/salt bridges are drawn as dashed lines in the 3D structures. The X-ray pose with PDB ID 6DE9 (Geldenhuys et al., 2019) is added to each frame.

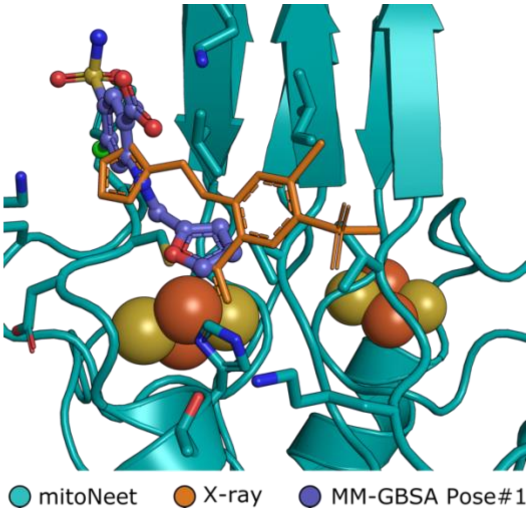

Figure SI13. The lowest energy MM-GBSA pose (purple ball and sticks, #1) as calculated with Schrödinger Suite (A). As all of the other top 10 poses, it differs largely from that in the X-ray structure (orange sticks, RMSD=8.9 Å) and calculated by metadynamics (RMSD ranges from 7.3 Å from **Ib**, to 9.4 Å from **Ic**, poses not shown). The pose which is similar to that in the X-ray pose, is ranked as #121.

These results support the usefulness of going beyond simple (yet fast) docking approaches to study the binding of furosemide to mitoNEET.

2.2 Supplementary Tables

Table SI1. Energies calculated with the Schrödinger MM-GBSA suit (Shivakumar et al., 2010) (see Section 1.2 and Figure SI13). The binding energies - much larger (in absolute value) than the experimental (Geldenhuys et al., 2016) and calculated values of the binding free energies (this work) - are used here to rank the poses.

Energies (kcal/mol)	Highest Ranked Pose	Pose #121 (X-ray-like)
Ligand Strain	1.9	0.5
MMGBSA ΔG Bind	-39.2	-23.3
MMGBSA ΔG Bind (NS)	-37.2	-22.7

Table SI2. H-bonds/salt bridges and hydrophobic contact distances in **I-III**.

	Lig. - Atom	Prot.- Res.	Prot.- Atom	Distance (Å)	Interaction
Pose Ia	O21	LYS55	NZ	2.9	Salt bridge
	O20	LYS55	NZ	2.9	Salt bridge
	O20	HIS87	NE2	2.8	H-bond
	O16	LYS68	NZ	3.0-2.8 (O16-OW-NZ)	water mediated H-bond
	C13	PRO100	CG	4.4	Hydrophobic
	C10	PRO100	CG	4.2	Hydrophobic
	C3	GLY85	CA	3.3	Hydrophobic
	C1	THR88	CG2	3.7	Hydrophobic
	C12	VAL70	CG1	5.1	Hydrophobic
	C11	ILE102	CD	4.7	Hydrophobic
Pose Ib	O20	LYS55	NZ	2.9	Salt bridge
	O21	LYS55	NZ	2.9	Salt bridge
	O20	HIS87	NE2	2.9	H-bond
	O18	C83	O	2.7-2.8 (O18-OW-O)	water mediated H-bond
	O20	T88	OG1	2.7-2.6 (O20-OW-OG1)	water mediated H-bond

	C1	VAL57	CG2	3.6	Hydrophobic
	C2	VAL57	CG2	4.0	Hydrophobic
	C3	VAL57	CG1	3.6	Hydrophobic
	C3	ILE102	CD	4.2	Hydrophobic
	C6	ILE102	CD	4.1	Hydrophobic
	C13	PRO100	CG	4.1	Hydrophobic
	C13	VAL70	CG1	4.4	Hydrophobic
	C8-C13	H87	CG-ND	5.2	π-π-Stack.
Pose Ic	O20	LYS55	NZ	2.8	Salt bridge
	O20	HIS87	NE2	3.3	H-bond
	O20	THR88	OG1	2.5	H-bond
	C1	PRO100	CG	4.4	Hydrophobic
	C1	ILE102	CD	4.4	Hydrophobic
	C2	PRO100	CG	4.4	Hydrophobic
	C2	ILE102	CD	4.2	Hydrophobic
	C2	VAL70	CG1	3.5	Hydrophobic
	C2	VAL70	CG2	4.1	Hydrophobic
	C3	VAL70	CG1	3.7	Hydrophobic
	C3	VAL70	CG2	4.4	Hydrophobic
	C8-C13	H87	CG-ND	5.3	π-π-Stack.
Pose IIa	O20	LYS68	NZ	2.8	Salt bridge
	O21	LYS104	NZ	2.9	Salt bridge
	C13	ILE102	CG1	3.8	Hydrophobic
	C6	ILE102	CG1	4.0	Hydrophobic
	C11	ALA59	CB	3.8	Hydrophobic
	C12	ALA59	CB	4.0	Hydrophobic
	C6	VAL70	CG2	4.1	Hydrophobic
	C3	VAL70	CG2	3.6	Hydrophobic
	C2	VAL70	CG2	3.9	Hydrophobic
	C1	VAL70	CG2	3.8	Hydrophobic
Pose IIb	O20	LYS55	NZ	2.7	Salt bridge
	O16	VAL57	N	3.2	H-bond
	O18	ASN53	ND2	3.0	H-bond
	O16	ASN53	OD1	2.7-2.8 (O16-OW-OD1)	water mediated H-bond
	C2	PRO54	CB	3.5	Hydrophobic
	C3	PRO54	CB	4.0	Hydrophobic
	C10	VAL57	CB	4.3	Hydrophobic
	C10	VAL57	CG2	3.6	Hydrophobic

Pose III	O18	VAL57	N	2.9	H-bond
	O18	VAL57	O	2.7<br>(O18-OW-O)	water mediated H-bond
	N17	VAL57	O	3.2-2.8<br>(N17-OW-O)	water mediated H-bond
	O16	LYS55	NZ	3.1-3.0<br>(O16-OW-NZ)	water mediated H-bond
	C3	PRO54	CD	4.2	Hydrophobic
	C6	PRO54	CD	3.5	Hydrophobic
	C6	PRO54	CG	4.4	Hydrophobic
	C10	PRO54	CD	4.4	Hydrophobic
	C13	PRO54	CD	4.0	Hydrophobic

Table SI3. Free binding energy calculated from LV-MetaD simulation (ΔG^0 is the standard binding free energy, ΔG_{MetaD} is the free energy difference computed via LV-MetaD, and ΔG_{corr} is the correction term) and emerging from affinity measurements in vitro (ΔG_{exp}).

ΔG_{MetaD} (kcal/mol)	ΔG_{corr} (kcal/mol)	ΔG^0 (kcal/mol)	ΔG_{exp} (kcal/mol) (Geldenhuys et al., 2016)
5.9±0.8	1.8	7.7±0.8	5.8

3 References

Bai, F., Morcos, F., Sohn, Y.-S., Darash-Yahana, M., Rezende, C.O., Lipper, C.H., et al. (2015). The Fe-S cluster-containing NEET proteins mitoNEET and NAF-1 as chemotherapeutic targets in breast cancer. *Proc. Natl. Acad. Sci. U.S.A.* 112(12)**,** 3698-3703. doi: doi:10.1073/pnas.1502960112.

Capelli, R., Carloni, P., and Parrinello, M. (2019). Exhaustive Search of Ligand Binding Pathways via Volume-Based Metadynamics. *J. Phys. Chem. Lett.* 10(12)**,** 3495-3499. doi: 10.1021/acs.jpclett.9b01183.

Geldenhuys, W.J., Long, T.E., Saralkar, P., Iwasaki, T., Nuñez, R.A.A., Nair, R.R., et al. (2019). Crystal structure of the mitochondrial protein mitoNEET bound to a benze-sulfonide ligand. *Commun. Chem.* 2. doi: 10.1038/s42004-019-0172-x.

Geldenhuys, W.J., Yonutas, H.M., Morris, D.L., Sullivan, P.G., Darvesh, A.S., and Leeper, T.C. (2016). Identification of small molecules that bind to the mitochondrial protein mitoNEET. *Bioorg. Med. Chem. Lett.* 26(21)**,** 5350-5353. doi: 10.1016/j.bmcl.2016.09.009.

Halgren, T.A., Murphy, R.B., Friesner, R.A., Beard, H.S., Frye, L.L., Pollard, W.T., et al. (2004). Glide: a new approach for rapid, accurate docking and scoring. 2. Enrichment factors in database screening. *Journal of Medicinal Chemistry* 47(7)**,** 1750-1759. doi: papers3://publication/doi/10.1021/jm030644s.

Sastry, G.M., Adzhigirey, M., Day, T., Annabhimoju, R., and Sherman, W. (2013). Protein and ligand preparation: parameters, protocols, and influence on virtual screening enrichments. *Journal of Computer-Aided Molecular Design* 27(3)**,** 221-234. doi: papers3://publication/doi/10.1007/s10822-013-9644-8.

Schrödinger, L.L.C. (2019a). "Schrödinger Release 2019-4: LigPrep". (New York, NY: Schrödinger,LLC).

Schrödinger, L.L.C. (2019b). "Schrödinger Release 2019-4: Protein Preparation Wizard". (New York: Schrödinger).

Shivakumar, D., Williams, J., Wu, Y., Damm, W., Shelley, J., and Sherman, W. (2010). Prediction of Absolute Solvation Free Energies using Molecular Dynamics Free Energy Perturbation and the OPLS Force Field. *Journal of Chemical Theory and Computation* 6(5)**,** 1509-1519. doi: 10.1021/ct900587b.

Wang, J., Wolf, R.M., Caldwell, J.W., Kollman, P.A., and Case, D.A. (2004). Development and testing of a general amber force field. *J. Comput. Chem.* 25(9)**,** 1157-1174. doi: 10.1002/jcc.20035.

Zhao, Q., Capelli, R., Carloni, P., Lüscher, B., Li, J., and Rossetti, G. (2021). Enhanced Sampling Approach to the Induced-Fit Docking Problem in Protein–Ligand Binding: The Case of Mono-ADP-Ribosylation Hydrolase Inhibitors. *J. Chem. Theory Comput.* 17(12)**,** 7899-7911. doi: 10.1021/acs.jctc.1c00649.
